# Temporal Feature Extraction and Machine Learning for Classification of Sleep Stages Using Telemetry Polysomnography

**DOI:** 10.3390/brainsci13081201

**Published:** 2023-08-14

**Authors:** Utkarsh Lal, Suhas Mathavu Vasanthsena, Anitha Hoblidar

**Affiliations:** 1 Department of Computer Science and Engineering, Manipal Institute of Technology, Manipal Academy of Higher Education, Manipal 576104, Karnataka, India; utkarsh.lal@learner.manipal.edu; 2 Department of Electronics and Communication Engineering, Manipal Institute of Technology, Manipal Academy of Higher Education, Manipal 576104, Karnataka, India; anitha.h@manipal.edu

**Keywords:** polysomnography, electroencephalography, electromyography, electrooculography, power spectral density, Higuchi fractal dimension, singular value decomposition entropy, permutation entropy, detrended fluctuation analysis, XGBoost

## Abstract

Accurate sleep stage detection is crucial for diagnosing sleep disorders and tailoring treatment plans. Polysomnography (PSG) is considered the gold standard for sleep assessment since it captures a diverse set of physiological signals. While various studies have employed complex neural networks for sleep staging using PSG, our research emphasises the efficacy of a simpler and more efficient architecture. We aimed to integrate a diverse set of feature extraction measures with straightforward machine learning, potentially offering a more efficient avenue for sleep staging. We also aimed to conduct a comprehensive comparative analysis of feature extraction measures, including the power spectral density, Higuchi fractal dimension, singular value decomposition entropy, permutation entropy, and detrended fluctuation analysis, coupled with several machine-learning models, including XGBoost, Extra Trees, Random Forest, and LightGBM. Furthermore, data augmentation methods like the Synthetic Minority Oversampling Technique were also employed to rectify the inherent class imbalance in sleep data. The subsequent results highlighted that the XGBoost classifier, when used with a combination of all feature extraction measures as an ensemble, achieved the highest performance, with accuracies of 87%, 90%, 93%, 96%, and 97% and average F1-scores of 84.6%, 89%, 90.33%, 93.5%, and 93.5% for distinguishing between five-stage, four-stage, three-stage, and two distinct two-stage sleep configurations, respectively. This combined feature extraction technique represents a novel addition to the body of research since it achieves higher performance than many recently developed deep neural networks by utilising simpler machine-learning models.

## 1. Introduction

On average, people spend a third of their 24 h day sleeping, making it an essential physiological process that significantly impacts an individual’s overall health [1]. Understanding sleep stages and their characteristics is essential for diagnosing and treating sleep disorders, affecting millions worldwide [2,3]. Sleep can be broadly classified into two primary stages: non-rapid eye movement (NREM) and rapid eye movement (REM) sleep. NREM is further divided into three distinct stages, characterised by different levels of brain activity and physiological responses. The five distinct stages of sleep identified in this study are as follows [4]:

1. Wake (W): Marked by low-amplitude, mixed-frequency brain waves, with normal muscle tone and high mentation.
2. Stage 1 (N1): First NREM stage. EEG shows low-voltage and mixed-frequency activity while eye movement and muscle activity begin to decrease.
3. Stage 2 (N2): Marked by sleep spindles and K-complexes in the EEG signal. Muscle tone, heart rate, and eye movement further slow down, and body temperature drops.
4. Stages 3 and 4 (N3/N4): Deepest stage of NREM. Crucial for physical restoration and memory consolidation. This stage is also known as the slow-wave sleep stage. EEG shows high-amplitude and low-frequency delta waves. There is minimal eye movement, and muscle tone is at its lowest during NREM sleep.
5. REM (R): Marked by rapid eye movement, dreaming, and temporary muscle paralysis to prevent physical stimulation from dreams. EEG shows low-amplitude and mixed-frequency activity, which is similar to the N1 stage.

Sleep stages N1 and N2 signify Light Sleep, while N3 and N4 signify Deep Sleep.

The accurate detection of sleep stages plays a pivotal role in both clinical and research settings. Clinically, the comprehensive characterisation of a patient’s sleep architecture, including the distribution and duration of the different sleep stages through the course of the night, is fundamental for diagnosing a diverse set of sleep disorders. For instance, disruptions in sleep stage patterns in a patient are integral diagnostic criteria for insomnia [5,6,7], sleep apnoea [8,9], and narcolepsy [10,11]. Moreover, sleep stage classification is not only essential for diagnosing these disorders but also for monitoring the efficacy of various treatments. Sleep stage detection enables clinicians to accurately assess the impact of treatments such as Continuous Positive Airway Pressure (CPAP) for sleep apnoea [12] or Cognitive Behavioural Therapy for insomnia (CBT-I) [13].

In the research setting, accurate sleep stage classification is pivotal for examining sleep’s impact on cognitive functions like learning and memory consolidation. Notably, slow-wave sleep (SWS) is crucial for memory consolidation and synaptic plasticity [14]. Clinically, sleep stage detection can aid in studying sleep’s correlations with ageing and neurodegenerative disorders like Alzheimer’s [15]. Hence, advancing sleep staging can enhance diagnostics and treatments for sleep and neurodegenerative issues.

However, the task of sleep stage detection has traditionally been performed manually by trained specialists analysing polysomnography (PSG) data. Recording these PSG data is a tedious task, wherein the subject has to stay overnight in the lab. Due to the presence of multiple modalities and long recording durations, accurately detecting sleep stages from PSG is a challenging task. On the other hand, modalities like actigraphy [16] present an easy alternative to record data that can be used to detect sleep stages using wearable devices, like wristbands or watches, that measure movement and activity levels over extended periods of time. However, the ease of recording sleep data using actigraphy presents an inherent tradeoff. Actigraphy can lack the degree of precision and reliability of the information captured, making it often less accurate than PSG [17]. Additionally, ECG and heart rate variability (HRV) can also be recorded to identify sleep stages. However, this approach does not provide any information regarding brain activity, which is a crucial aspect of sleep stage analysis [18]. Since the data’s accuracy and consistency are paramount to creating a model that can effectively differentiate between sleep stages, PSG data have been chosen as the modality in this study. With its multimodal nature, PSG captures fine physiological details, like eye and muscle movements and brain wave changes, with high temporal resolution. Furthermore, sleep stage classification with PSG adheres to standardised criteria from systems like the American Academy of Sleep Medicine (AASM) and Rechtschaffen and Kales (R&K), ensuring consistency across different studies and clinical settings.

Due to the extensive nature of polysomnography (PSG) data, which includes hours-long recordings of multiple signals such as electroencephalography (EEG), electromyography (EMG), and electrooculography (EOG), processing this vast amount of data can be challenging. The complexity arises from the numerous sources of voltage fluctuations captured within the recordings, making direct data processing quite demanding. Therefore, feature extraction methods must be employed to accurately capture the subtleties and minute variations in all of these signals in order to uniquely identify different sleep stages. Although previous research has been conducted on multiple feature extraction measures, an exploratory study highlighting the comparative analysis of all the feature extraction measures reported in this paper is yet to be reported. We aim to fill this research gap by presenting an in-depth performance analysis of multiple measures drawn from different categories, such as entropy, fractal dimensions and spectral analysis features. We also extract statistical features like the mean, standard deviation, kurtosis, and skewness from all bio-signals in the PSG data. This in-depth feature extraction and machine-learning approach is a novel strategy that has not been deeply explored previously for the specific measures explored in this study.

Although extracting relevant features is a critical step, having a mechanism that can learn from these features and accurately distinguish between various sleep stages is equally crucial. In recent studies, deep neural networks have been widely employed for feature extraction and classification [19]. However, these neural networks can get highly complex and require longer training times than simpler statistical and machine-learning models. Additionally, neural networks are intrinsically black-box models, which makes it challenging to interpret the results yielded by the model.

In light of these drawbacks, we explore machine-learning techniques in this study instead of neural networks to highlight the efficacy of straightforward machine-learning models in classifying sleep stages when trained on an enriched set of features that capture salient biomarkers of sleep stages from PSG data. This approach can potentially circumvent the need for complex deep-learning models for the classification of sleep stages.

In the initial phase of this study, the following machine-learning models were evaluated to establish baseline performance metrics—Extreme Gradient Boosting, Light Gradient Boosting Machine, Random Forest, K-Nearest Neighbours, Linear Discriminant Analysis, Quadratic Discriminant Analysis, Support Vector Machines, Naive Bayes, Ridge Classifier, and Extremely Randomized Trees. These models were trained on 80% of the ensemble feature data and tested on the remaining 20%. Table 1 presents the baseline metrics of these models. The top four models demonstrating the highest performance metrics were selected for further use in this study:

- Extreme Gradient Boosting (XGBoost);
- Light Gradient Boosting Machine (LGBM);
- Random Forest (RF);
- Extremely Randomized Trees/Extra Trees (ET).

Notably, all these algorithms are ensemble methods based on decision trees. XGBoost and LGBM are boosting models, wherein each new tree is built one at a time. On the other hand, RF and ET are bagging models, wherein many trees are independently built, and their predictions are averaged. Therefore, bagging and boosting algorithms illustrated superior performance to others in recognising the key differentiating characteristics of different sleep stages from the extracted features. Therefore, based on the rationale of choosing explicit feature extraction for capturing salient biomarkers for sleep staging, coupled with straightforward machine-learning models for the predictive task, the following objectives were designed for this study:

- To develop a model that combines an optimal feature extraction method with an optimal supervised learning model to distinguish between the different sleep stages with high accuracy.
- To evaluate the performance of the following feature extraction measures:
  – Power spectral density (PSD);
  – Singular value decomposition (SVD) entropy;
  – Higuchi fractal dimension (HFD);
  – Permutation entropy (PE);
  – Detrended fluctuation analysis (DFA);
  – Mean;
  – Standard deviation (Std Dev);
  – Kurtosis;
  – Skewness.
- To evaluate the performance of this model in accurately distinguishing between various sleep stages in the following configurations:
  – Five sleep stages, consisting of Wake (W), N1, N2, N3/4, and REM (R);
  – Four sleep stages, consisting of W, Light Sleep, Deep Sleep, and R;
  – Three sleep stages, consisting of W, Non-REM, and REM;
  – Two sleep stages, consisting of Non-REM and REM;
  – Two sleep stages, consisting of Wake (W) and Sleep (Non-REM and REM).

The objectives are framed to answer the following Research Question (RQ):

RQ: *To what extent do SVD entropy, PSD, HFD, PE, DFA, mean, Std Dev, kurtosis, and skewness, as feature extraction measures, differentiate between the different stages of sleep?*

The subsequent sections of this article are structured as follows. In Section 2, we thoroughly analyse existing sleep stage detection approaches, summarising the most recent breakthroughs in this domain. Then, Section 3 delineates our adopted methodology and design strategy, which involves a rigorous examination of various feature extraction and machine-learning approaches for creating an optimal pipeline that is capable of effectively differentiating between various sleep stage configurations using polysomnography (PSG) data. Subsequent to this, Section 4 presents our research findings, followed by Section 5, where we compare the performance of our model with other recent works performed in the same domain. Afterwards, in Section 6, we delve into a detailed discussion of the potential reasons behind the results observed in our study. The paper concludes with Section 7, where we accentuate the unique contributions of this study to the existing body of knowledge and present the key takeaways and insights gathered from our explorations.

## 2. Related Work

Multiple studies have been conducted that employ different methods for detecting sleep stages, ranging from state-of-the-art machine-learning models that distinguish between sleep stages based on features extracted from PSG data to complex deep-learning models [20] designed to work independently without the need for any explicit feature extraction or dimensionality reduction techniques. In one such study, a one-dimensional convolutional neural network (CNN) was developed to detect sleep stages directly from the raw data with high accuracy [21]. Another study [22] implemented a 1-D CNN model to detect cyclic alternating patterns (CAPs) in the EEG data, achieving an accuracy of 90.46% for classifying three-class sleep stages. Studies like [23] proposed a light and efficient deep neural network model based on fractional Fourier transform (FRFT) features derived from the EEG signal, which achieved an accuracy of 81.6% in sleep stage classification. Other studies like [24] developed a novel non-contact sleep structure prediction system (NSSPS) using radio-frequency signals and a convolutional recurrent neural network. This study achieved accuracies ranging from 66 to 83% for classifying various sleep stages. One of the notable contributions to the body of research was the SleepEEGNet [25] presented in 2019. This study utilised the EEG signal and implemented a combination of a Recurrent Neural Network (RNN) sequence-to-sequence model with a CNN for classifying sleep stages. This study used the same dataset (PhysioNet Sleep-EDF) employed in our study and achieved an overall accuracy of 84.26%.

The PhysioNet Sleep-EDF dataset is one of the most widely utilised datasets for sleep analysis. Multiple studies have analysed this dataset for sleep stage classification using neural networks. One such study [26] developed an automated system for sleep stage classification using a deep convolutional long short-term neural network (CNN-LSTM). Another similar study [27] on the same dataset employed a CNN-LSTM model for sleep stage classification using a single-channel Fpz-Cz EEG channel and achieved an accuracy of 84.19%. Single-channel EEG is one of the most popular choices of modalities for sleep stage analysis, and multiple studies, like [28], have been conducted for sleep stage classification. However, the utilisation of EMG and EOG data along with EEG using a diverse spectrum of feature extraction measures, like DFA, entropy, fractal dimensions, PSD, and statistical measures, has not been deeply explored yet.

Deep-learning models achieve high accuracy; however, such models often take a long time to train and test due to the sheer volume of PSG data, which are often recorded over long durations for each subject. In order to reduce the complexity and increase the efficiency of a predictive model, there is a need to extract the salient features from PSG data that capture the nuances and principal characteristics that mark the differences between the various sleep stages without any loss of information. Numerous methods have been implemented in previous research to efficiently extract features by using measures such as fractal dimensions [29], entropy measures [30], wavelet transforms [31], and power spectral density [32].

Although PSG data are one of the most widely accepted modalities for sleep analysis, recent technological developments have opened up new avenues that are much easier for the same task. With the surge of sleep-tracking devices, such as smart watches, smart rings, and other actigraphy-based devices, a new phase of sleep stage detection research is underway. Many studies have been conducted to compare the performance of the popular consumer-grade product Oura Ring with PSG data when recorded simultaneously. In one such study, sleep-onset latency (SOL), total sleep time (TST), and wake after onset (WASO) were computed and compared from the recordings of both PSG and Oura Ring [33]. Multiple discrepancies were observed between PSG and Oura, indicating a need for further enhancement of such consumer products in order to increase their overall accuracy. In a study bearing significant similarity, physiological data gathered through both the Oura Ring and polysomnography (PSG) were employed in an examination of various sleep-related factors. Specifically, the study focused on exploring the influence of peripheral signals mediated by the autonomic nervous system (ANS), circadian characteristics, and accelerometer data on sleep stage detection [34]. The Oura Ring includes a triaxial accelerometer, a negative temperature coefficient (NTC) thermistor as a temperature sensor, and an infrared photodetector that measures heart rate variability (HRV). The research indicated that combining the small size of wearable ring technology, multidimensional biological data streams, and effective artificial intelligence algorithms can result in notable precision in discerning sleep stages.

Apart from wearable rings, wristwatch-type sensing devices have also been employed for sleep stage detection in previous studies. In one such study, a combination of a reflective photoelectric volume pulse sensor and a triaxial accelerometer was utilised for sleep quality assessment and compared with PSG data, recorded simultaneously [35]. An analysis of pulse-to-pulse (PPI) and body movement indexes derived from the physiological signals recorded by the wristwatch sensor were used to develop an automated sleep stage classification system. In other studies, novel approaches like non-contact radar technology have also been implemented to accurately distinguish between sleep stages [36].

While many studies utilise multiple modalities of data and multidimensional biometric streams, as discussed previously, some studies focus on heart rate variability (HRV) for the classification of sleep stages and even other disorders. In one such study, detrended fluctuation analysis (DFA) and spectral analysis were employed to quantify cyclical variation related to the heart to investigate the effect of sleep stages and sleep apnea on HRV [37]. Similarly, another study utilises the Firstbeat sleep analysis method, which is based on measurements derived from HRV and accelerometer data [38].

## 3. Materials and Methods

### 3.1. Dataset

The PhysioNet Sleep-EDF expanded (sleep-edfx) dataset was utilised in this study [39,40]. The dataset contains two different studies:

1. Sleep Cassette (SC): This project focused on analysing age-related effects on sleep in a healthy Caucasian demographic ranging from 25 to 101 years old, with participants abstaining from any sleep-related medications. This investigation conducted two successive polysomnography (PSG) recordings, each spanning approximately 20 h, during consecutive day–night cycles at the participants’ residences. The components of each PSG incorporated electrooculography (EOG), electroencephalography (EEG), and submental electromyography (EMG) signals. While the EEG and EOG data were sampled at a rate of 100 Hz, the EMG data were gathered at 1 Hz. Furthermore, parameters such as oro-nasal airflow, rectal body temperature, and event markers were logged at a frequency of 1 Hz. The recording of the PSG data was facilitated by a device akin to a Sony Walkman cassette.
2. Sleep Telemetry (ST): This study investigated temazepam medication effects on sleep in 22 Caucasian men and women with no other medications. The subjects complained of having mild difficulty falling asleep but had no other conditions. Nine-hour PSG recordings were conducted in a hospital for two nights. Prior to recording PSG, temazepam was administered to the patient on one of the nights, and a placebo was given on the other. EOG, EMG, and EEG signals were sampled at 100 Hz with an event marker at 1 Hz.

Furthermore, the dataset also contains a hypnogram containing expert annotations of sleep stages for each PSG recording. In this study, PSG obtained from the ST study was utilised. Since two sessions were conducted for each of the 22 subjects, there were a total of 44 sessions, with each session having two files, one each for PSG and the hypnogram.

All files are present in the European Data Format (.edf). Each recording contains EEG from Fpz-Cz and Pz-Oz electrode locations, EOG, and EMG recorded from the chin and event markers.

Expert technicians manually labelled the hypnograms in accordance with the Rechtschaffen and Kales standard [41]. The different labels used to denote classes in the dataset include Wake (W), N1 (Stage 1), N2 (Stage 2), N3 (Stage 3), N4 (Stage 4), M (movement time), and “?” (not scored).

### 3.2. Preprocessing

**Figure 1:**
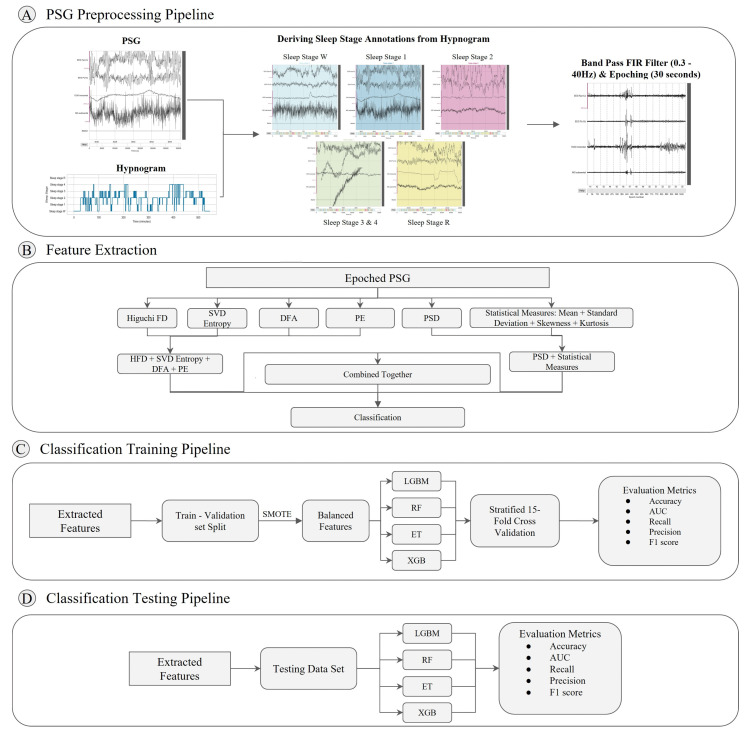
Illustration of the architecture designed for sleep stage detection. (**A**) PSG preprocessing pipeline consisting of the following steps: annotation of PSG sleep stages from hypnogram, band pass filtering, and epoching. (**B**) Robust features are extracted from the epoched PSG using various feature extraction measures. Different combinations of these measures were employed with classifiers to find the optimal model. (**C**) Training pipeline incorporating Synthetic Minority Oversampling Technique (SMOTE) for rectifying class imbalance and stratified K-fold cross-validation across 15 folds. (**D**) Testing pipeline utilising imbalanced dataset for evaluating trained classifiers.

For preprocessing, the MNE and NumPy Python libraries were employed. Figure 1A presents an overview of the preprocessing pipeline implemented in this study.

**Figure 2:**
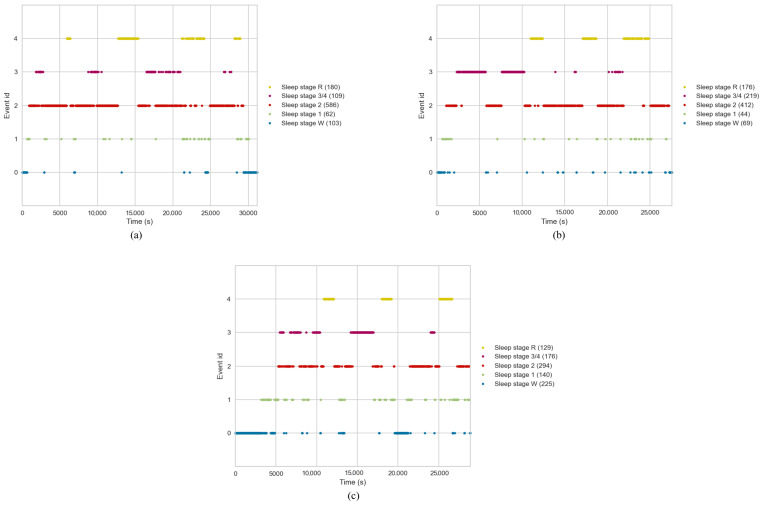
Sleep stage distribution plot. (**a**) Patient A, (**b**) Patient B, (**c**) Patient C.

- **Data ingestion**: Raw PSG files were loaded using the MNE python package [42]. A Finite Impulse Response (FIR) filter was implemented to perform filtering with a high-pass filter of 0.3 Hz to reduce low-frequency drift and a low-pass filter of 40 Hz. The Firwin method was used for this filter, which incorporates a Hamming window with 0.0194 passband ripple and 53 dB stopband attenuation. Raw hypnogram files were loaded using the MNE package and integrated with the PSG data as annotations. These annotations provided information about the different sleep stages and their durations in the time domain.
- **Sleep stage mapping**: Sleep stages were mapped to different configurations as 5-stage (Wake, N1, N2, N3/N4, REM), 4-stage (Wake, Light, Deep, REM), 3-stage (Wake, Non-REM, REM), 2-stage (REM, NREM), and another 2-stage (Wake and REM). Figure 2 shows the distribution of these sleep stages with respect to time for 3 different subjects. Each event was mapped to an Event ID, as shown in the figure. As is evident, Wake stages are skewed towards the left, which is the initial part of the sleep cycle, while the REM stages are skewed towards the right, indicating their occurrence towards the later phases of the sleep cycle. Furthermore, sleep stage 2 has the highest number of events, indicating that the average individual spends the most amount of time in Light Sleep. Stages 3 and 4 were combined into one class named N3/N4. Furthermore, classes M and ‘’?” were excluded. Execution of the whole pipeline was conducted for each configuration, making a total of 6 different runs.
- **Event and epoch extraction**: After defining the sleep stage mapping configuration, the PSG data were segmented into epochs, each representing a 30 s window. Using the MNE package, the annotations were converted into events. Based on these events, epochs were extracted from the PSG data, wherein each epoch was labelled with the corresponding sleep stage.
- **SMOTE**: An imbalance in the classes of the target variable (i.e., sleep stage) was observed in the dataset, wherein the number of events for the different sleep stages was highly varied. This issue became more pronounced in 2-stage and 3-stage sleep classification tasks, where multiple classes were integrated. To balance the dataset, the Synthetic Minority Oversampling Technique (SMOTE) was employed [43].

### 3.3. Feature Extraction

A set of feature extraction measures was computed for every epoch. Figure 1B represents the steps taken for extracting features using various measures. While most studies employ entropy and fractal dimension measures to extract features from EEG signals, this study also employed the standard deviation, skewness, and kurtosis as statistical feature extraction measures from EOG and EMG to capture all relevant features from each bio-signal in PSG data. A brief overview of every feature extraction method employed in this study is given below.

#### 3.3.1. Power Spectral Density

**Figure 3:**
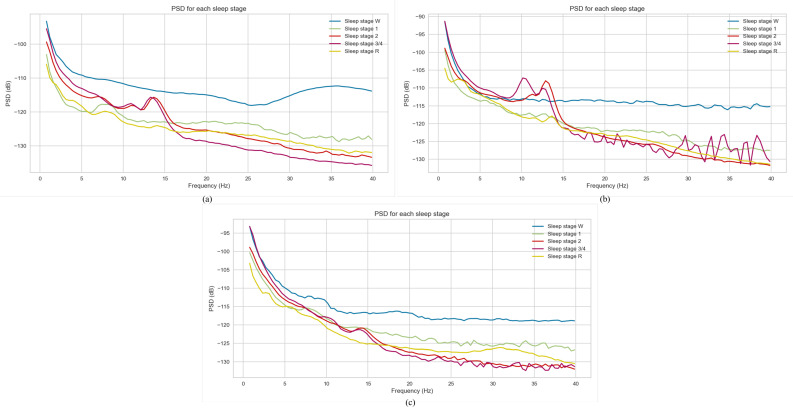
Power spectral density plot. (**a**) Patient A, (**b**) Patient B, (**c**) Patient C.

Power spectral density (PSD) is a measure that provides information about the distribution of signal power across a range of frequencies. By applying PSD, more information about dominant frequencies in the PSG data can be gained. In this study, Welch’s method [44] was employed to compute PSD for each epoch of the PSG signal. Figure 3 represents the power spectral density plot of three subjects from the dataset utilised in this work. For all three plots, the Wake sleep stage exhibits the highest power spectral density, with Deep Sleep (N3/N4 stages) having the lowest values. For calculating PSD, in the first step, the input signal—x(0)),x(1),…x(N−1), where N is the number of data points—is segmented into K partitions or batches.

For every partition (k = 1 to K), the Discrete Fourier Transform (DFT) is computed at a frequency of v=i/M with −(M/2−1)≤i≤M/2 [44]:

(1) $$X_{k}\left(v\right)=\sum_{m}x\left(m\right)w\left(m\right)exp\left(-j*2\pi vm\right)$$

where m=(k−1)S,…M+(k−1)S−1, and w(m) is the window function. For each segment (k = 1 to K), a new value called the periodogram value, $P_{k}\left(f\right)$, is computed from the DFT:

(2) $$P_{k}\left(v\right)=\frac{1}{W}{\left|X_{k}\left(v\right)\right|}^{2}$$

where

(3) $$W=\sum_{m=0}^{M}w^{2}\left(m\right)$$

Then, Welch’s PSD is computed by taking the average of the values for every periodogram.

(4) $$S_{x}\left(v\right)=\frac{1}{K}\sum_{k=1}^{K}P_{k}\left(v\right)$$

Welch’s method is also known as the periodogram averaging method. To summarise, the input time series is divided into successive blocks, after which periodograms are formed for each block using Equation (2). The values of periodograms across time give the value for PSD.

#### 3.3.2. Singular Value Decomposition (SVD) Entropy

SVD entropy is a measure that quantifies the intricacy or uncertainty present in time-series data. It is rooted in the singular value decomposition algorithm, a mathematical approach that decomposes a matrix into three distinct matrices revealing the hidden patterns in the data. It employs eigenvectors to provide an accurate representation of the data. As the complexity of the data increases, more orthogonal vectors are necessary to represent it effectively, which consequently leads to a higher SVD entropy value.

For an input signal, $\left[x_{1},x_{2},\ldots x_{n}\right]$, the first delay vectors are defined as follows:

(5) $$y\left(i\right)=\left[x_{i},x_{i+\tau },\ldots x_{i+(d_{E}-1)\tau }\right]$$

where τ and $d_{E}$ are the delay and embedding dimension, respectively. A $d_{E}$ of 3 and a τ of 1 were used in this study. Afterwards, the embedding space is constructed by:

(6) $$Y={\left[y_{1},y_{2},\ldots y_{n-(d_{E}-1)\tau }\right]}^{T}$$

The SVD entropy is then defined as follows [45,46,47]:

(7) $$H_{SVD}=-\sum_{i=1}^{M}{\bar{\sigma }}_{i}\log_{2}\left({\bar{\sigma }}_{i}\right)$$

where M is the number of singular values of an embedded matrix Y, and $\sigma_{1},\sigma_{2},\ldots \sigma_{M}$ are the normalised singular values such that ${\bar{\sigma }}_{i}=\sigma_{i}/\sum_{j=1}^{M}\sigma_{j}$.

#### 3.3.3. Higuchi Fractal Dimension (HFD)

Introduced by T. Higuchi in 1988, this technique generates multiple time series by systematically sub-sampling the signal into individual time series [48]. For each new time series, the length of the signal curve is calculated, averaged across all samples, and subsequently plotted on a logarithmic graph. The slope of this graph signifies the Higuchi FD value of the signal. In the first step, for a signal with the data points x(1), x(2), x(3), …x(n), Higuchi’s technique generates a new time series for each value of k (K = 1, 2, 3 …$k_{max}$), where k represents the interval length or time duration. For each time series, the signal is sampled at intervals of size k.

For each of these new signals, the length of the signal is calculated as:

(8) $$L_{m}\left(k\right)=\frac{\sum_{i=1}^{\left\lfloor \frac{N-m}{k}\right\rfloor }|X\left(m+ik\right)-X\left(m+\left(i-1\right)\right)k|y}{k}$$

where y represents the normalisation factor as:

(9) $$y=\frac{\left(N-1\right)}{\lfloor \frac{N-m}{k}\rfloor k}$$

The length of the time series for the interval k, denoted by L(k), is computed as the mean value across k groups of $L_{m}\left(k\right)$ as follows:

(10) $$L\left(k\right)=\frac{\sum_{i=1}^{k}L_{m}\left(k\right)}{k}$$

The slope of the linear least-squares fit of log(L(k)) plotted against log(1/k) gives the Higuchi FD. Consequently, the mean across k groups of $L_{m}\left(k\right)$, computed as L(k) in Equation (10), follows the power law given by:

(11) log(L(k))∝D.log(1/k),

and then the time series has the fractal dimension D [49]. A default $k_{max}$ = 10 was taken for this study’s calculation of the Higuchi FD.

#### 3.3.4. Permutation Entropy

PE is based on finding a signal’s ordinal patterns (also known as permutations). The ordinal pattern of a tuple of m real numbers $\left(x_{1},x_{2},\ldots x_{m}\right)$ provides information about the relationship between its elements. PE quantifies the complexity of the signal by capturing the order relations between values of the signal and extracting a probability distribution of ordinal patterns [50].

In this study, for every epoch consisting of a time series of length D, the segments w(i)=w(i1),w(i2),w(i3),⋯,w(iD) are extracted from the original time series:

(12) $$x_{1},x_{2},x_{3},\cdots ,x_{N}$$

by taking consecutive components $x_{i}$, which can be separated by a time delay or latency τ as:

(13) $$\tau :x_{i},x_{i+\tau },x_{i+2\tau },\ldots ,x_{i+(D-1)\tau }$$

The recommended value of D ∈[3,7] and τ=1 [50]. Consequently, a sequence of D-length vectors is formed from the original time series using the delay τ. For each D-length vector, ordinal patterns are created by ranking the data points in the vector. The occurrence of each possible ordinal pattern is counted in the entire series and normalised to obtain the relative frequency of each ordinal pattern, after which the permutation entropy is computed as follows:

(14) $$PE_{D}=-\sum_{i=1}^{D!}p_{i}log_{2}p_{i}$$

where D! is the total number of possible ordinal patterns, and $p_{i}$ is the relative frequency.

#### 3.3.5. Detrended Fluctuation Analysis

DFA is a statistical technique used for analysing non-stationary time-series data. It evaluates long-range correlations and self-similarity properties within the data. DFA is founded on examining the fluctuation behaviour of a time series after removing local trends at different time scales, which helps assess the scaling properties of the data and determine the absence or presence of long-range correlations [51].

For a time series $\left[x_{1},x_{2},\ldots x_{N}\right]$, the procedure to compute DFA is as follows:

1. First, x is integrated into series y=[y(1),…y(N)], where $y\left(k\right)=\sum_{i=1}^{k}\left(x_{i}-\bar{x}\right)$, and $\bar{x}$ is the mean of $x_{1},x_{2},\ldots x_{N}$.
2. The integrated series is then divided into segments of equal length n, such that in each segment, a least-squared line is fit to the data, which represents the trend within that segment. The y-coordinate of these linear segments is labelled $y_{n}\left(k\right)$.
3. Using the equation:
  (15) $$F\left(n\right)=\sqrt{\left(1/N\right)\sum_{k=1}^{N}{\left[y\left(k\right)-y_{n}\left(k\right)\right]}^{2}}$$
  the variation in the root-mean-square of the integrated series is calculated. The part $y\left(k\right)-y_{n}\left(k\right)$ in the equation is called **detrending.**
4. Lastly, the fluctuation is calculated by computing the slope of the line relating log(F(n)) to log(n).

#### 3.3.6. Statistical Time-Domain Features

The following statistical measures were computed for every 30 s epoch in the signal and used as features for the classification task.

- Mean
  (16) $$Mean=\frac{\mathrm{Sum}\;\mathrm{of}\;\mathrm{the}\;\mathrm{values}\;\mathrm{of}\;\mathrm{data}\;\mathrm{points}\;\mathrm{in}\;\mathrm{the}\;\mathrm{epoch}}{\mathrm{Total}\;\mathrm{number}\;\mathrm{of}\;\mathrm{data}\;\mathrm{points}\;\mathrm{in}\;\mathrm{the}\;\mathrm{epoch}}$$
- Standard Deviation
  (17) $$\begin{array}{ccc} \sigma & = & \sqrt{\frac{\sum {\left(x_{i}-\mu \right)}^{2}}{N}}\\ where\\ \sigma & = & \mathrm{standard}\;\mathrm{deviation};\\ N & = & \mathrm{the}\;\mathrm{length}\;\mathrm{or}\;\mathrm{total}\;\mathrm{data}\;\mathrm{points}\;\mathrm{in}\;\mathrm{the}\;\mathrm{epoch};\\ x_{i} & = & \mathrm{each}\;\mathrm{data}\;\mathrm{point}\;\mathrm{in}\;\mathrm{the}\;\mathrm{epoch};\\ \mu & = & \mathrm{mean}\;\mathrm{of}\;\mathrm{all}\;\mathrm{data}\;\mathrm{points}\;\mathrm{in}\;\mathrm{the}\;\mathrm{epoch}. \end{array}$$
- Skewness
  (18) $$\begin{array}{ccc} \tilde{\mu_{3}} & = & \frac{\sum_{i}^{N}{\left(X_{i}-\bar{X}\right)}^{3}}{\left(N-1\right)*\sigma^{3}}\\ where\\ \tilde{\mu_{3}} & = & \mathrm{skewness};\\ N & = & \mathrm{total}\;\mathrm{number}\;\mathrm{of}\;\mathrm{data}\;\mathrm{points}\;\mathrm{in}\;\mathrm{the}\;\mathrm{epoch};\\ X_{i} & = & \mathrm{each}\;\mathrm{data}\;\mathrm{point}\;\mathrm{in}\;\mathrm{the}\;\mathrm{epoch};\\ \bar{X} & = & \mathrm{mean}\;\mathrm{of}\;\mathrm{the}\;\mathrm{distribution};\\ \sigma & = & \mathrm{standard}\;\mathrm{deviation}. \end{array}$$
- Kurtosis
  (19) $$\begin{array}{ccc} Kurt & = & \frac{\mu_{4}}{\sigma^{4}}\\ where\\ \mu_{4} & = & \mathrm{Fourth}\;\mathrm{Central}\;\mathrm{Moment};\\ \sigma^{4} & = & \mathrm{standard}\;\mathrm{deviation}. \end{array}$$

### 3.4. Classification

After feature extraction, for PSD, the output was a 3-dimensional list of the following dimensions: number of epochs, number of channels, and number of frequencies, where the number of epochs was 41,490, and the number of channels was 4, namely, EEG Fpz-Oz, EEG Pz-Oz, EOG horizontal and EMG submental. The initial list was flattened and converted into a dataframe of the following dimensions: number of epochs, number of channels * number of frequencies, where the number of channels * number of frequencies is equal to 405. Therefore, the final input table consisted of 405 columns and 41,490 rows of data that were fed into the classifiers, wherein each row signified the features extracted from a particular epoch. For SVD Ent, HFD, PE, and DFA, the input table consisted of the 4 PSG channels as columns, namely, EEG Fpz-Cz, EEG Pz-Oz, EOG horizontal, and EMG submental, and 41,490 rows, wherein each row contained the 4 features computed for each epoch. For statistical measures, the input table consisted of 16 columns. For each of the 4 PSG channels, 4 statistical measures, namely, mean, standard deviation, skewness, and kurtosis, were calculated. After feature extraction, the number of rows for all tables was equal to the total number of epochs, i.e., 41,490.

Supervised multi-class classification was performed on the features extracted using the previously mentioned feature extraction measures. The following machine-learning algorithms were employed for this task:

- Extreme Gradient Boosting (XGBoost);
- Light Gradient Boosting Machine (LGBM);
- Random Forest Classifier (RF);
- Extra Trees Classifier (ET).

The data were divided into training and testing sets comprising 80% and 20% of the entire dataset, respectively. For the training dataset, stratified K-fold cross-validation was conducted across K = 15 folds, wherein the subset was divided into 15 segments of equal size, out of which 14 segments were used for training the model and one segment was used for validating the performance of the model. This process was repeated 15 times with different combinations of training and validating segments, and metrics across all 15 folds were averaged to give the final result. Afterwards, the model was tested on unseen and imbalanced testing data that were held out of the training process, wherein SMOTE was not applied.

A total of five classification tasks were conducted in this study as follows:

1. Five-stage sleep detection: distinguishing between Wake, N1, N2, N3/4, and REM stages;
2. Four-stage sleep detection: distinguishing between Wake, Light (N1 + N2), Deep (N3 + N4), and REM stages;
3. Three-stage sleep detection: distinguishing between Wake, Non-REM (N1 + N2 + N3 + N4), and REM stages;
4. Two-stage sleep detection (a): distinguishing between Non-REM (N1 + N2 + N3 + N4) and REM stages;
5. Two-stage sleep detection (b): distinguishing between Wake (W) and Asleep (N1 + N2 + N3 + N4 + REM).

### 3.5. Evaluation Metrics

- Recall
  (20) $$Recall=\frac{TruePositives}{TruePositives+FalseNegatives}$$
- Precision
  (21) $$Precision=\frac{TruePositives}{TruePositives+FalsePositives}$$
- Accuracy
  (22) $$Accuracy=\frac{TP+TN}{TP+TN+FP+FN}*100$$
  (23) $$TPR=\frac{TruePositives}{TruePositives+FalseNegatives}$$
  (24) $$FPR=\frac{FalsePositives}{FalsePositives+TrueNegatives}$$

## 4. Results

### 4.1. Five-Stage Sleep Classification

#### 4.1.1. Individual Performance of Feature Extraction Measures

Initially, all feature extraction measures were employed separately to assess their individual performance in distinguishing sleep stages in the five-stage configuration having W, N1, N2, N3/N4, and R sleep stages, as described in Section 3.4.

Table 2 presents the average metrics (Accuracy, Recall, Precision, AUC) across 15 folds of cross-validation for all individual feature-extraction–classifier combinations that were implemented for five-stage sleep detection. Statistical measures consisted of the mean, skewness, standard deviation, and kurtosis, which were computed for every epoch of every subject’s PSG data and concatenated horizontally in a tabular format. PSD yielded the highest accuracy with the XGB classifier: 82.25%. Statistical measures also yielded good individual performance with the highest accuracy of 82.17% with RF. However, SVD entropy, HFD, PE, and DFA achieved subpar accuracies.

**Figure 4:**
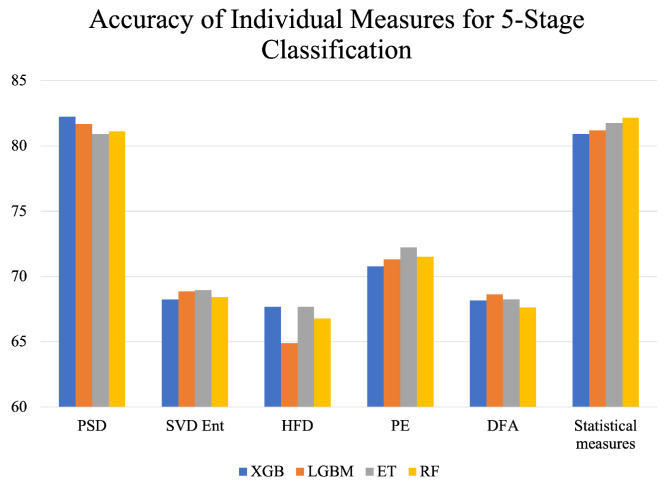
Accuracy of individual feature extraction measures across different classifiers for 5-stage classification.

Figure 4 represents a visual comparison of the performance of different feature extraction measures. It can be observed that HFD gives the lowest accuracies across all classifiers, indicating the inefficacy of HFD in accurately capturing the features of sleep that can distinguish between the different sleep stages. PSD performs the best, followed by the statistical measures PE, DFA, SVD Ent, and HFD, in decreasing order of accuracy. The performance of classifiers varied for different feature extraction measures. For PSD, XGB achieved the highest accuracy but the lowest accuracy for statistical measures. This can be attributed to differences in the nature of the feature extraction measures and different dimensions of the input tables, as described in Section 3.4 of this study. Precision, recall, and AUC were in line with the patterns followed by the accuracy metric for all feature extraction measures shown in Table 2.

#### 4.1.2. Combined Performance of Feature Extraction Measures

After conducting experimentation for sleep stage detection using one feature extraction method at a time, as evident from Table 2 and Figure 4, it was found that the accuracy, precision, and recall are suboptimal for all classifiers since they are below 88%. Therefore, for further experimentation, multiple measures were combined together to enrich the data and aid in better classification of the five sleep stages.

The following combinations of feature extraction measures were employed in this study:

- SVD Ent, HFD, DFA, and PE;
- PSD and statistical measures (i.e., mean, standard deviation, skewness, and kurtosis);
- PSD, SVD Ent, HFD, DFA, and PE;
- All combined together (PSD, SVD Ent, HFD, DFA, PE, mean, standard deviation, skewness, and kurtosis).

For creating the above combinations, the individual features extracted from the PSG were concatenated horizontally, creating a wider input to the classifiers with a large number of features to learn from.

Table 3 presents the average accuracies across 15 folds of cross-validation for all coalesced feature-extraction–classifier combinations that were implemented to distinguish between the five sleep stages, namely, W, N1, N2, N3/N4, and REM. The highest accuracy was given by the model that consisted of all feature extraction measures combined together with the XGB Classifier. This model achieved an accuracy of 87.44%. The lowest accuracy was obtained by the combination of PSD and statistical measures with the RF classifier.

**Figure 5:**
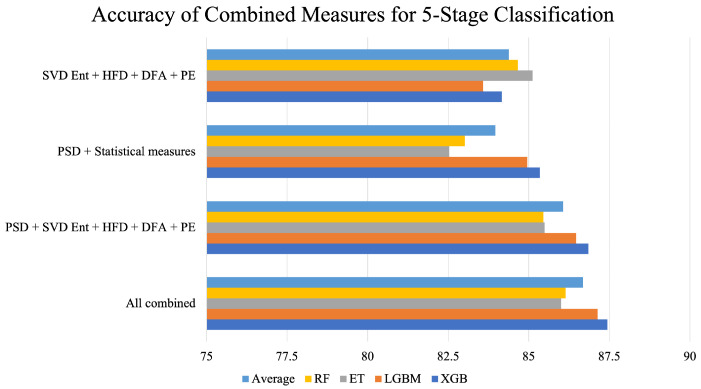
Accuracy of combined feature extraction measures across different classifiers for 5-stage classification. The bar “Average” represents the mean of RF, ET, LGBM, and XGB for every combination of feature extraction measures.

Figure 5 sheds more light on the performance of the combined feature extraction measures. PSD with statistical measures yielded the lowest average accuracy, while the combination of all measures performed the best. It can also be observed from the figure that, as more measures were combined, an incremental rise in accuracy was achieved.

The best performance was achieved by integrating all feature extraction measures for the five-stage sleep classification. As a result, this effective approach was also employed for the four-stage, three-stage, and two-stage sleep classification tasks.

Moreover, for the most effective classification model, the feature table was partitioned into training and testing subsets at a respective ratio of 80:20. Notably, the Synthetic Minority Oversampling Technique (SMOTE) was excluded from the application to the testing set, thereby resulting in a class imbalance. The model, once trained, was then evaluated on these unseen and imbalanced testing data.

**Figure 6:**
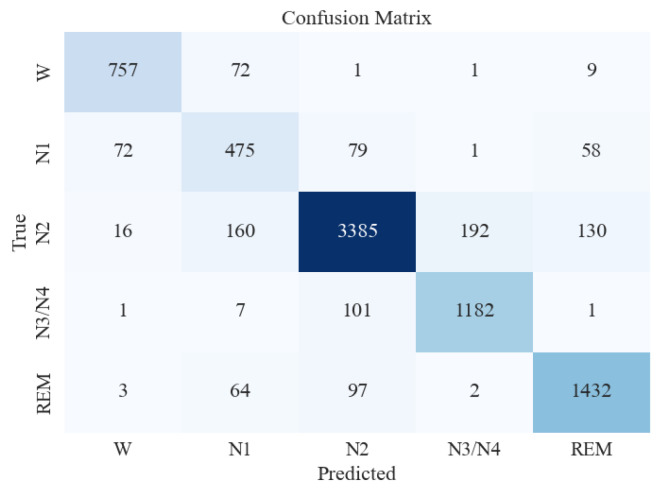
Confusion matrix for XGBoost classifier for 5-stage sleep classification using all feature extraction measures together.

Figure 6 presents the confusion matrix obtained from the XGB classifier when features were extracted using all measures combined. This confusion matrix represents the model’s performance on an unseen and imbalanced testing dataset. The *y*-axis represents the actual/true values, and the *x*-axis represents the predicted values of various sleep stages. The number of misclassifications for W is extremely low, whereas it is mildly higher between N1, N2, and N3/N4. Table 4 presents the classification report of the XGB classifier on testing data. This process was conducted to understand the model’s performance on imbalanced and unseen data. The Support column in Table 4 represents the total number of instances of a particular sleep stage in the testing data. As is evident, the class imbalance is high. However, the model yielded a good accuracy of 87%. High precision was observed with all classes except the N1 sleep stage, for which the model yielded poor metrics. This may be attributed to the extremely low number of data instances for N1 in the testing (in Table 4, support for N1 = 685).

### 4.2. Four-Stage Sleep Classification

In four-stage sleep classification, models were used to distinguish between four sleep stages, namely, Wake (W), Light (N1 + N2), Deep (N3 + N4), and REM (R). Table 5 presents the average metrics obtained from 15-fold cross-validation for the four-stage sleep classification. As evident from the table, XGB achieves the highest accuracy of 90.08% and the highest recall of 91.32%.

**Figure 7:**
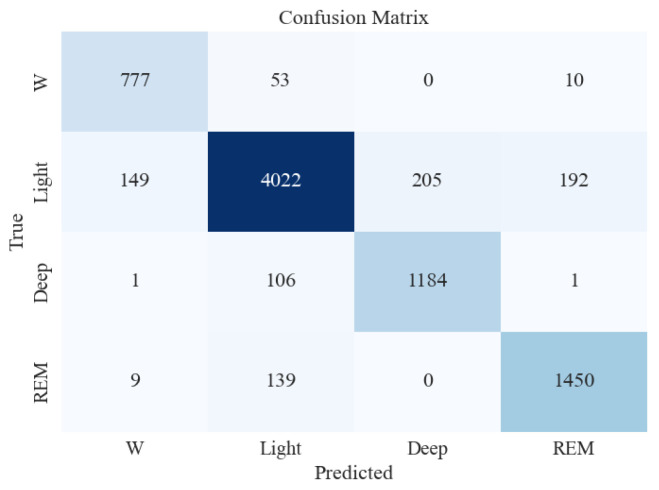
Confusion matrix for XGBoost classifier for 4-stage sleep classification using all feature extraction measures together.

Similar to the five-stage sleep classification, the model that gave the best accuracy (XGB) was tested on unseen and imbalanced testing data to evaluate its performance further. Table 6 and Figure 7 present the classification report and confusion matrix obtained from the testing set.

From Table 6, it can be seen that the accuracy on the testing dataset increased to 90% in the four-stage sleep classification from 87% in the five-stage sleep classification. The precision for N1 and N2 also increased by a big margin to 93%. Overall, the performance improved when moving from five-stage to four-stage sleep classification.

### 4.3. Three-Stage Sleep Classification

In three-stage sleep classification, models were used to distinguish between three sleep stages, namely, Wake (W), Non-REM (N1, N2, N3/N4), and REM (R). Table 7 presents the average metrics obtained from 15-fold cross-validation for the three-stage sleep classification.

**Figure 8:**
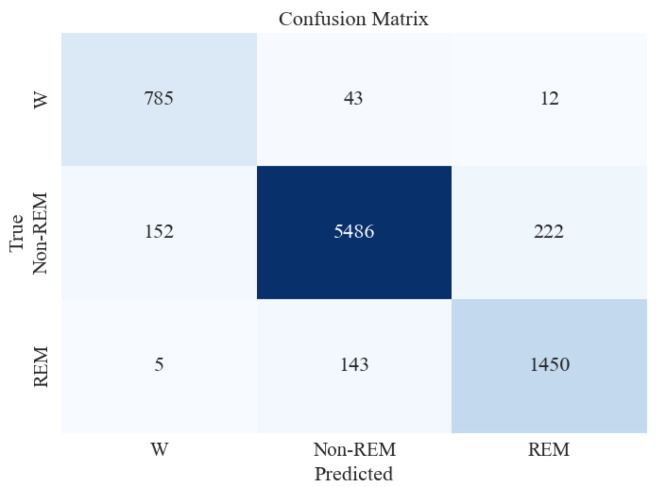
Confusion matrix for XGBoost classifier for 3-stage sleep classification using all feature extraction measures together.

Similar to the five-stage sleep classification, the XGB classifier was tested on unseen and imbalanced testing data to evaluate its performance further. Table 8 and Figure 8 present the classification report and confusion matrix obtained from the model’s performance on the testing set.

Figure 8 shows that the accuracy of testing data increased from 87% in the previous section to 93%. Precision and recall for the Non-REM class are very high, followed by those of REM and Wake. This can be attributed to a large number of data instances for the Non-REM class, as evident in the Support column of the figure.

### 4.4. Two-Stage Sleep Classification

In two-stage sleep classification, the models were used to distinguish between two sleep stages in two different sleep stage configurations as follows:

- Distinguishing between Non-REM (N1, N2, N3, N4) and REM (R);
- Distinguishing between Awake (W) and Asleep (N1, N2, N3, N4, R).

The following subsections provide results for these two classification tasks.

#### 4.4.1. REM vs. Non-REM

**Figure 9:**
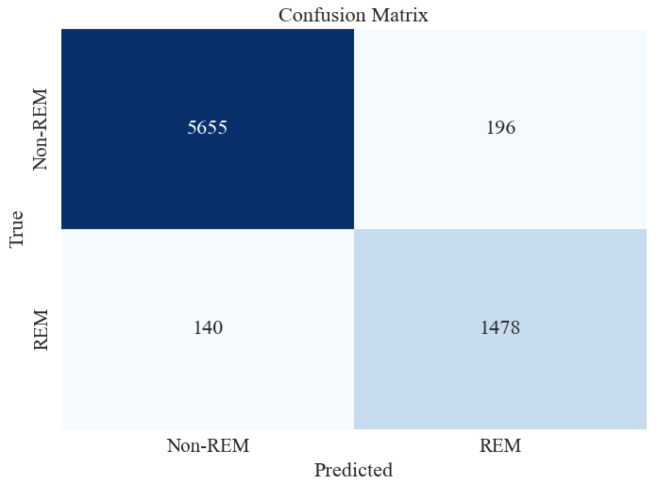
Confusion matrix for XGBoost classifier for 2-stage (Non-REM vs. REM) sleep stage classification using all feature extraction measures together.

Table 9 presents the average metrics obtained from 15-fold cross-validation for the two-stage (NREM vs. REM) sleep classification. Similar to the five-stage sleep classification, XGB was tested on unseen and imbalanced testing data to evaluate its performance further. Table 10 and Figure 9, respectively, present the classification report and confusion matrix obtained from the testing set.

Figure 9 shows that the accuracy of testing data increased from 93% in the previous section to 96%. Precision and recall for the Non-REM class are very high, followed by those of REM. This may be attributed to a large number of data instances for the Non-REM class, as evident in the Support column of the figure.

#### 4.4.2. Awake vs. Asleep

**Figure 10:**
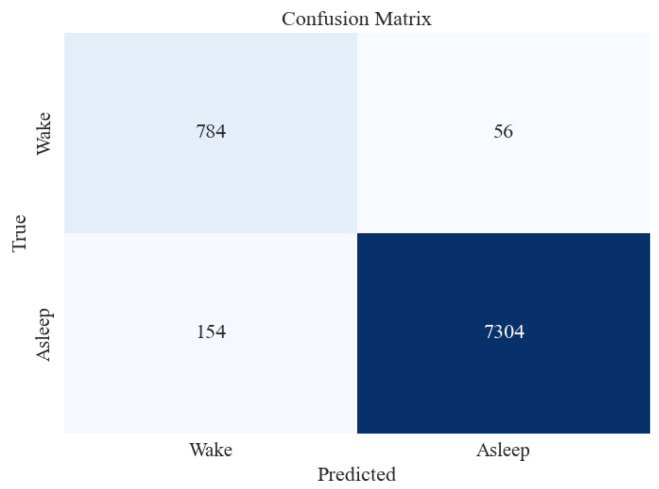
Confusion matrix for XGBoost classifier for 2-stage (Awake vs. Asleep) sleep classification using All feature extraction measures together.

Table 11 presents the average metrics obtained from 15-fold cross-validation, for the two-stage (Awake vs. Asleep) sleep classification. Similar to the five-stage sleep classification, XGB was tested on unseen and imbalanced testing data to evaluate its performance further. Table 12 and Figure 10, respectively, present the classification report and confusion matrix obtained from the testing set.

Figure 10 shows that the accuracy of testing data increased from 96% in the previous section to 97%. Precision and recall for the “Asleep” class are very high, followed by those of “Awake”. This may be attributed to a large number of data instances for the “Asleep” class, as evident in the Support column of the figure.

## 5. Comparison with Recent Works

The comparative analysis of recent significant studies on sleep stage detection is summarised in Table 13. This table incorporates thirteen notable studies, eight of which utilised the PhysioNet Sleep EDF Database for their investigations. Our research also leverages the same database, specifically the Expanded Sleep-EDF Database of 2018 (notated as Sleep-EDF-18).

The examination of Table 13 reveals a clear inclination towards EEG signals derived from PSG as the primary modality for sleep analysis. This preference stems from the capacity of EEG to capture vital brain activity during sleep, offering definitive and discernible biomarkers for different sleep stages. In contrast, EOG and EMG provide information about eye and muscle movement and are often very noisy [60,61], which problematises distinguishing between the Non-REM sleep stages. However, in spite of their shortcomings, EOG and EMG signals provide valuable information for identifying the REM sleep stage [62] since they are accompanied by rapid eye movement captured by EOG and muscle atonia, which EMG can capture. Therefore, the use of all three modalities in a judicious way can enhance the performance of models for accurately differentiating between different sleep stages.

Yet, this integrated approach presents its own set of challenges. The task of processing and extracting key biomarkers from all three signals is inherently more complex than working with the EEG signal alone. This intricacy is evident in Table 13, where studies that have employed multiple PSG modalities along with EEG often struggle to achieve high classification accuracies, even after implementing sophisticated deep-learning models.

As observed from the table, refs. [24,53,54] utilised multiple modalities along with EEG from the PhysioNet Sleep-EDF dataset, which has also been used for our paper. It is evident that the ensemble feature extraction method used in our study, coupled with a straightforward machine-learning model like XGBoost, achieved superior results to several deep convolutional neural networks. This highlights the significance of feature extraction methods for enriching data quality and thereby considerably increasing the predictive performance of models.

Furthermore, multiple studies, such as [23,24,52,53,54], either employed a single method of feature extraction or multiple measures having similarity in their functioning, as observed in [52], which used multiple measures that are similar to each other to some degree. On the other hand, methods like [57] employed a diverse set of features along with a machine-learning approach, which, in turn, achieved better performance than most of the other studies. This indicates that measures having varied natures of functioning can complement each other and uniquely identify relevant features from the signals. For instance, fractal dimensions and entropies primarily elucidate the complexity and irregularity inherent in brain activity, whereas the power spectral density analysis emphasises the distribution of power across distinct frequency components within the signal. Concurrently, Hjorth parameters provide information regarding the signal’s power, its mobility (mean frequency), and its intrinsic complexity. The integration of these diverse methods, supplemented by statistical measures such as kurtosis and skewness, affords a more comprehensive approach to feature extraction. Kurtosis and skewness measure the asymmetry of the probability distribution about the mean, along with more information about outliers and peak sharpness. This approach can provide a comprehensive feature extraction method that captures relevant features in a multifaceted manner.

In a deeper examination of studies that exclusively utilised the EEG signal, it can be observed that several research efforts have employed intricate, deep neural networks to secure high accuracies, as is evident in Table 13 in [22,26,57]. But interestingly, some studies, such as [58], have focused on extensive feature extraction, employing the tunable Q-factor wavelet transform (TQWT) and Normal Inverse Gaussian (NIG) Probability Density Function modelling to capture the principal differentiating characteristics for sleep stage classification, thereby enhancing the quality of the input data for the predictive models. When processed through an Adaptive Boosting model (AdaBoost), these refined data yielded an impressive accuracy rate of 94% for sleep stage classification. In contrast to this study, which focused on EEG data, our study utilised all bio-signals from PSG data (i.e., EEG, EMG, and EOG). Additionally, our study followed a similar feature extraction approach but explored measures of a different nature, including fractal dimension, spectral, entropy, and statistical features. This exploratory study gives a fresh perspective on sleep stage classification by investigating a diverse spectrum of feature extraction measures. Furthermore, this approach precludes the need for a deep-learning model since it achieves higher accuracies than many complex deep neural networks implemented previously. Thus, we posit that a focus on sophisticated feature extraction could potentially be a more efficient path towards improving sleep stage classification performance.

## 6. Discussion

We aimed to find the optimal model consisting of a feature extraction measure and machine-learning algorithm that can accurately differentiate between different sleep stages in PSG data. For this purpose, different feature extraction measures and their combinations were employed with different classifiers to assess the individual as well as the grouped performance of the measures. The results shed light upon the efficacy of an ensemble method that combines all measures together as an effective feature extraction technique that accurately captures relevant information from the multiple signals—EEG, EMG, and EOG—present in PSG data. Amongst all methods implemented in this study, the most effective method for sleep stage detection consists of the following techniques:

- Feature extraction: A combination of PSD, SVD entropy, HFD, DFA, PE, and statistical measures, namely—mean, standard deviation, skewness, and kurtosis.
- Classifier: Extreme Gradient Boosting (XGBoost).

The accuracies yielded for different sleep stage configurations for the above model are as follows:

- Five-stage sleep classification (W, N1, N2, N3/N4, R): 87.44%;
- Four-stage sleep classification (W, Light Sleep N1, N2, Deep Sleep N3, N4, R): 90.08%;
- Three-stage sleep classification (W, Non-REM N1, N2, N3, N4, REM R): 93.31%;
- Two-stage sleep classification (Non-REM, REM): 95.34%;
- Two-stage sleep classification (Awake W, Asleep N1, N2, N3, N4, R): 97.34%.

**Figure 11:**
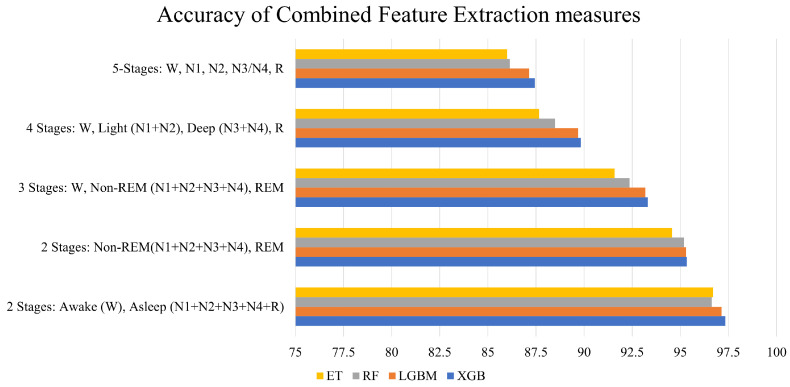
Accuracy of combined feature extraction measures for each sleep stage configuration.

From Figure 11, it can be observed that the performance of all classifiers increases when the number of sleep stages to be distinguished decreases. As evident in the figure, for five-stage sleep detection, the performance of all classifiers was the lowest compared to all other sleep stage configurations, while the performance for two-stage sleep detection was the highest. However, between the two distinct two-stage sleep classification configurations (i.e., Non-REM vs. REM and Awake vs. Asleep), each classifier achieved greater accuracy in differentiating between Wake (W) and Asleep (N1, N2, N3, N4, R) stages than in the case of Non-REM vs. REM, as evident from Figure 11. This may be attributed to the possibility that all Non-REM and REM sleep stages have some intrinsic characteristics in common [63], which makes differentiating between Non-REM and REM sleep stages more difficult than differentiating between Sleep and Wake states since the Wake state is associated with distinctly more movement, muscle activity, and brain function than the sleep state.

Furthermore, it can also be observed in Figure 4 that feature extraction measures, when employed individually, do not achieve good performance. In particular, the Higuchi fractal dimension, which measures the complexity or the degree of intricacy of the signal, could not capture relevant EEG features for differentiating between the various sleep stages since it gave the lowest accuracy when employed individually. Amongst SVD Ent, HFD, PE, and DFA, PE performed better by a considerable margin since it gave an accuracy of 72%. In contrast, the other measures peaked at around 68% for the different classifiers implemented. However, among all individual measures, the highest accuracies were achieved by PSD, as evident from Figure 4, closely followed by statistical measures. The reason behind this can be attributed to the different nature of these feature extraction measures as well as the greater number of features extracted for the same epoch. To substantiate further, PSD measures the power distribution of the signal across different frequency bands, which implies that multiple features were extracted for the same epoch corresponding to different signal frequencies. Therefore, the number of features for PSD (405, as mentioned in Section 4) was much higher than that for HFD, DFA, ZCR, or PE (four each, as mentioned in Section 4). Similarly, the number of features for the statistical measures was 16 (4 for each—mean, standard deviation, skewness, and kurtosis). Having a greater number of features enabled the classifiers to learn from more data instances and thus capture the nuances of PSG data better than HFD, DFA, ZCR, and PE individually.

With respect to the performance of various machine-learning models, it is evident from Figure 11 that the XGBoost classifier outperforms all other classifiers in each of the sleep stage configurations, while the Extra Trees classifier gives the lowest accuracy in most configurations.

Lastly, a significant observation that is clear from Figure 5 is that as more measures were combined together one by one, the performance of all classifiers increased. For example, when HFD, SVD Ent, DFA, and PE were combined, all classifiers achieved accuracies over 83%. In contrast, their individual performance was limited to an accuracy of 62–72%, as evident in Table 2. Similarly, when these measures were combined with PSD, the accuracies of all classifiers were over 85%, as evident in Table 3. This indicates that each feature extraction measure is able to capture unique features that other measures cannot. Therefore, each measure uniquely aids in detecting sleep stages by identifying salient features from the PSG data. Since PSG consists of multiple signals—EOG, EMG, and EEG, which, respectively, record eye movement, muscle movement, and brain activity—it is clear that the data being captured by the signals differ in nature. Therefore, it is possible that a few feature extraction measures, like PSD, might capture relevant features for accurately differentiating between sleep stages from the EEG signal but may fail to accurately capture them from the EMG signal. Meanwhile, another feature extraction method may complement PSD in capturing salient features from the EMG signal that helps differentiate between the different sleep stages. There is scope to further strengthen this study by exploring this avenue for determining the specific ways in which the feature extraction measures uniquely aid in sleep stage detection while complementing each other.

The central premise of our study underscores the pivotal role of efficient feature extraction, utilising measures that inherently differ in their nature of functioning. This approach ensures the capture of a wide spectrum of characteristics from the polysomnography (PSG) signals, namely, EEG, EMG, and EOG, thereby providing a more comprehensive understanding of sleep stage dynamics. An approach like this enriches the data fed into predictive models and precludes the need for training complex deep neural networks that require immense development effort and overhead in the form of time and resources. This study achieved high accuracy, precision, recall, and F1-score by employing an extensive feature extraction approach coupled with a straightforward boosting machine-learning model (XGBoost).

Furthermore, a simpler and more efficient mechanism that automates the process of sleep staging has the potential to ease clinical diagnostics and research in sleep disorders such as insomnia, sleep apnoea, and narcolepsy. Therefore, we hypothesise that focusing on sophisticated feature extraction could be an efficient alternative to complex deep-learning approaches for improving sleep stage classification performance.

However, one of the limitations of our proposed model is its lack of interpretability. Further research can be conducted to understand which feature extraction measures uniquely capture which features from EEG, EOG, and EMG signals present in the PSG data. Adding this layer of interpretability to the architectural pipeline will shed more light on the unique contribution of each feature extraction measure for sleep stage classification. Moreover, there is scope to further strengthen this study by testing the proposed model on sleep datasets that follow guidelines given by the American Academy of Sleep Medicine (AASM) for sleep scoring. The PhysioNet sleep EDF Database, which was utilised in this study, employs the Rechtschaffen and Kales (R & K) guidelines for sleep scoring. The widespread usage of this database in recent studies allows our work to be directly comparable with these studies and their proposed models. Additionally, our study can also be readily compared with studies that utilise other sleep databases, such as the Cyclic Alternating Pattern (CAP) [64] and the University College Dublin Sleep Apnea (UCD) [65] datasets, which also employ the R & K sleep scoring system. However, the robustness of the model constructed in this study can be further validated by testing its performance on other datasets following different sleep-scoring protocols.

## 7. Conclusions

There is a research gap in the comparative analysis of feature extraction measures, like the power spectral density (PSD), singular value decomposition (SVD) entropy, Higuchi fractal dimension (HFD,) detrended fluctuation analysis (DFA) and permutation entropy (PE), with respect to sleep stage detection. This study aims to fill this research gap. Furthermore, while recent studies on sleep stage detection have utilised PSG data by developing complex neural networks, we aim to highlight the efficacy of a simpler architectural pipeline comprising a novel temporal feature extraction technique that combines several existing measures together and an effective machine-learning model. From the results given in Section 4 of this paper, it can be observed that an ensemble feature extraction method consisting of PSD, SVD entropy, HFD, DFA, and PE coupled with statistical measures—mean, standard deviation, skewness, and kurtosis—can effectively capture salient features that can distinguish between sleep stages for all different sleep stage configurations. It was also observed that the XGBoost classifier performed consistently better than all other classifiers employed in this study. For five-stage, four-stage, three-stage, two-stage (NREM vs. REM), and another two-stage (Awake vs. Asleep) sleep classification, accuracies of 87.44%, 90.08%, 93.31%, 95.34, and 97.34% were, respectively, achieved using the XGBoost model, along with respective average F1-scores of 84.6%, 89%, 90.33%, 93.5%, and 93.5%. These results illustrate the high performance of a simpler architecture, which achieves accuracy rates higher than many previously built complex deep-learning models. Therefore, the results reveal an alternative research avenue that focuses on sophisticated feature extraction as a potentially more efficient and more straightforward path towards improving sleep stage classification performance.

## Figures and Tables

**Table 1:** Baseline metrics of all models initially tested. Top four models finally chosen for this study.

Model	Accuracy	AUC	Recall	Prec.	F1
XGBoost	0.8744	0.9793	0.8547	0.8777	0.8755
LGBM	0.8714	0.9791	0.8561	0.8764	0.873
RF	0.8614	0.9743	0.8396	0.8661	0.863
ET	0.86	0.9732	0.8329	0.8612	0.8601
SVM	0.7922	0.9112	0.7954	0.8166	0.7979
KNN	0.786	0.9295	0.794	0.8227	0.794
LDA	0.7688	0.9387	0.7622	0.7957	0.7771
Ridge	0.7401	0.8988	0.7493	0.7739	0.7459
QDA	0.4053	0.781	0.5103	0.6533	0.3153
NB	0.3388	0.7714	0.4021	0.5742	0.2118

**Table 2:** Results for 5-stage sleep classification with individual feature extraction measures.

Feature Extraction Measure	Classifier Model	Accuracy	AUC	Recall	Precision
Statistical measures	RF	82.17	96.45	78.84	83.51
	ET	81.75	96.19	77.28	82.84
	LGBM	81.2	96.14	76.25	82.21
	XGB	80.93	96.07	77.32	82.4
PSD	XGB	82.25	95.99	79.1	82.63
	LGBM	81.67	95.92	79.21	82.47
	RF	81.12	95.4	78.12	81.46
	ET	80.9	95.18	79.96	81
HFD	ET	67.69	88	64.66	68.06
	RF	66.79	88	65.07	67.86
	LGBM	64.9	88.94	67.26	68.69
	XGB	67.69	88	64.66	68.06
SVD entropy	ET	68.97	88.76	63.76	69.63
	LGBM	68.88	89.77	66.67	71.21
	XGB	68.25	89.53	65.96	70.71
	RF	68.43	88.67	64.18	69.77
PE	ET	72.22	91.01	68.71	73.08
	RF	71.52	90.91	68.92	72.86
	LGBM	71.33	91.93	71.7	74.23
	XGB	70.78	91.72	71	73.73
DFA	LGBM	68.64	89.51	66.25	71.03
	ET	68.26	88.25	63.08	69.04
	XGB	68.16	89.29	66.03	70.77
	RF	67.63	88.14	63.37	69.06

**Table 3:** Classification results for 5-stage sleep classification upon combining multiple feature extraction measures.

Feature Extraction Measure	Classifier Model	Accuracy	AUC	Recall	Precision
PSD + statistical measures	XGB	85.34	97.22	82.25	85.59
	LGBM	84.95	97.17	82.37	85.39
	RF	83.02	96.29	79.85	83.35
	ET	82.53	95.94	78.79	82.73
HFD, PE, DFA, SVD Ent	ET	85.12	97.03	82.66	85.3
	RF	84.66	96.87	83	85.18
	XGB	84.16	96.99	83.48	85.15
	LGBM	83.58	96.89	83.44	84.81
PSD, HFD, PE, DFA, SVD Ent	XGB	86.85	97.78	85	87.19
	LGBM	86.47	97.72	85.27	87.04
	ET	85.49	97.17	82.67	85.6
	RF	85.45	97.24	83.7	85.89
All combined	XGB	87.44	97.93	85.47	87.77
	LGBM	87.14	97.91	85.61	87.64
	RF	86.14	97.43	83.96	86.61
	ET	86	97.32	83.29	86.12

**Table 4:** Classification report of XGB classifier on testing data for 5-stage sleep classification when using all feature extraction measures together.

Sleep Stage	Precision	Recall	F1-Score	Support
W	89	90	90	840
N1	61	69	65	685
N2	92	87	90	3883
N3/N4	86	91	89	1292
R	88	90	89	1598
Accuracy			**87**	

**Table 5:** Results for 4-stage sleep classification upon combining all feature extraction measures.

Classifier	Accuracy	AUC	Recall	Precision
XGB	90.08	97.95	91.94	90.33
LGBM	89.69	97.77	91.32	90.01
RF	88.48	97.17	90.05	88.93
ET	87.66	96.9	89.21	88.11

**Table 6:** Classification report of XGB classifier on testing data for 4-stage sleep classification when using all feature extraction measures together.

Sleep Stage	Precision	Recall	F1-Score	Support
W	83	93	88	840
Light (N1/N2)	93	88	91	4568
Deep (N3/N4)	85	92	88	1292
R	88	91	89	1598
Accuracy			**90**	

**Table 7:** Results for 3-stage sleep classification upon combining all feature extraction measures.

Classifier	Accuracy	AUC	Recall	Precision
XGB	93.31	98.59	93.04	93.61
LGBM	93.18	98.57	93.22	93.56
RF	92.36	98.07	92.34	92.88
ET	91.58	97.83	91.59	92.16

**Table 8:** Classification report of XGB classifier on testing data for 3-stage sleep classification when using all feature extraction measures together.

Sleep Stage	Precision	Recall	F1-Score	Support
W	83	93	88	840
Non-REM (N1, …N4)	97	94	95	5860
REM (R)	86	91	88	1598
Accuracy			**93**	

**Table 9:** Results for 2-stage (Non-REM vs. REM) sleep classification upon combining all feature extraction measures.

Classifier	Accuracy	AUC	Recall	Precision
XGB	95.34	98.76	91.45	87.68
LGBM	95.29	98.76	92.41	87.07
RF	95.19	98.54	88.98	89.21
ET	94.56	98.23	86.74	88.43

**Table 10:** Classification report of XGB classifier on testing data for 2-stage sleep classification (NREM vs. REM) when using all feature extraction measures together.

Sleep Stage	Precision	Recall	F1-Score	Support
Non-REM	98	97	97	5851
REM	88	91	90	1618
Accuracy			**96**	

**Table 11:** Results for 2-stage (Awake vs. Asleep) sleep classification upon combining all feature extraction measures.

Classifier	Accuracy	AUC	Recall	Precision
XGB	97.34	99.47	97.77	99.27
LGBM	97.14	99.46	97.48	99.33
RF	96.63	99.25	96.83	99.4
ET	96.7	99.34	96.9	99.43

**Table 12:** Classification report of XGB classifier on testing data for 2-stage (Awake vs. Asleep) sleep classification when using all feature extraction measures together.

Sleep Stage	Precision	Recall	F1-Score	Support
Awake	84	93	88	840
Asleep	99	98	99	7458
Accuracy			**97**	

**Table 13:** Comparison of performance obtained by our model with other recent works.

Study	Year	Dataset	Classification Method	Feature Extraction Method	Modality	Overall Accuracy
**This study**	2023	PhysioNet Sleep-EDF-18	XGBoost	SVD entropy, Higuchi FD, DFA, PE, PSD, statistical measures	PSG	90.1% (4-stage), 93.34% (3-stage)
Zan et al. [52]	2023	PhysioNet Sleep-EDF-13	CNN	1-D Local Binary Patterns, Local Neighbour Descriptive Pattern, Local Gradient Pattern, Local Neighbor Gradient Pattern	PSG	84.80%
Jin et al. [53]	2023	PhysioNet Sleep-EDF-18, UCD and CAP	GCN and BiGRU	Short-Time Fourier Transform	PSG	80.07%
Kwon et al. [54]	2022	ISRUC	CNN	Multi-taper Spectrogram and CNN	PSG	81.52%
Zhai et al. [24]	2022	Original Dataset and MIT dataset	CRNN	CRNN	PSG and Radio Frequency	79.20%
Arslan et al. [55]	2022	Original Dataset	DNN	General preprocessing and undersampling	PSG	91.60%
Loh et al. [22]	2022	Cyclic Alternating Pattern (CAP) dataset	CNN	No explicit feature extraction	EEG	90.46%
Cvetko et al. [26]	2022	PhysioNet Sleep-EDF-13 and Sleep-EDF-18	CNN-LSTM	CEEMDAN, FFT, and PE	EEG	90.43%
You et al. [23]	2022	PhysioNet Sleep-EDF-13 Montreal Archive of Sleep Studies (MASS)	Bidirectional LSTM	Fractional Fourier Transform	EEG	81.60%
Zhang et al. [56]	2020	Not Open Access	Orthogonal-CNN	Hilbert–Huang transform,	EEG	88.40%
Zhou et al. [57]	2020	PhysioNet Sleep-EDF-13 and Sleep-EDF-18	Ensemble ML model— Random Forest + LGBM	Standard deviation, spectral entropy, Kraskov entropy, Renyi entropy, Hjorth parameters, Katz FD, Petrosian FD, Maximum-Minimum Distance, Hurst exponent and log root sum of sequential variations	EEG	91.20%
Mousavi et al. [25]	2019	PhysioNet Sleep-EDF-13 and Sleep-EDF-18	CNN and Bi-RNN	CNN and Bi-RNN	EEG	84.26%
Hassan et al. [58]	2017	PhysioNet Sleep Edf and St. Vincent’s University Hospital/University College Dublin Sleep Apnea Database	AdaBoost	TQWT and NIG Probability Density Function	EEG	94%
Tripathi et al. [59]	2022	PhysioNet Sleep-EDF-13	Ensemble ML	PSD, Pan-Tompking method for HRV extraction	ECG	96% (2-stage)

GCN: Graph Convolutional Network; BiGRU: Bidirectional Gated Recurrent Unit; CEEMDAN: Complete Ensemble Empirical Mode Decomposition; TQWT: Tunable-Q Factor Wavelet Transform; NIG: Normal Inverse Gaussian.

## Data Availability

The PhysioNet Sleep-EDF Expanded [39,40] (also known as sleep-edfx) dataset was utilised in this study. It can be found at this link—https://www.physionet.org/content/sleep-edfx/1.0.0/ (accessed on 28 June 2023).
